# *Borrelia mayonii* induces carditis but not arthritis in Lyme-susceptible mice

**DOI:** 10.3389/fimmu.2026.1727413

**Published:** 2026-01-22

**Authors:** Dawn W. Cleveland, Nivanthi Wijetunga, Timothy Casselli, Yvonne Tourand, Heidi L. Pecoraro, Catherine A. Brissette

**Affiliations:** 1 Department of Biomedical Sciences, University of North Dakota, Grand Forks, ND, United States; 2 Veterinary Diagnostic Laboratory, North Dakota State University, Fargo, ND, United States

**Keywords:** *Borrelia mayonii*, carditis, lyme arthritis, lyme disease, spirochetemia

## Abstract

**Background:**

*Borrelia mayonii*, a *Borrelia burgdorferi* sensu lato (Bbsl) genospecies, is a cause of Lyme disease in the upper midwestern United States. *B. mayonii* infection can present with some atypical clinical manifestations, including unusually high spirochetemia. Previous studies have demonstrated the infectivity of *B. mayonii* in laboratory mice and found evidence of widespread dissemination to internal tissues, however, did not report evidence of high spirochetemia. In the current study, we sought to directly compare spirochetemia as well as typical Lyme disease pathology including arthritis and carditis in Lyme disease-susceptible mice infected with either *B. mayonii* or *B. burgdorferi*.

**Methods:**

Immunocompetent (C3HeB/FeJ) and immunodeficient (SCID) mice were infected with *B. burgdorferi* or *B. mayonii*. Spirochetemia was measured in blood by qPCR, and pathology of hearts and joints examined.

**Results:**

Consistent with previous reports, we found that *B. mayonii* spirochetemia observed in human patients is not recapitulated in C3H mice and did not reach higher detectable levels compared to *B. burgdorferi*. Interestingly, tibiotarsal joint swelling and histopathology were restricted to *B. burgdorferi*-infected mice and were not detected in any *B. mayonii*-infected mice up to four weeks post-inoculation. Carditis was routinely detectable in both *B. mayonii* and *B. burgdorferi*-infected mice, although differences in kinetics and severity were observed.

**Conclusion:**

Taken together, this study characterizes similarities and differences between *B. burgdorferi* and *B. mayonii* in a laboratory model of Lyme disease. These findings add to the immunopathologic landscape caused by distinct genospecies in the Bbsl complex, which could shed light on the distinct host-pathogen interactions important for specific Lyme disease manifestations.

## Introduction

*Borrelia mayonii* is a novel causative agent of Lyme disease (LD) that was discovered in 2016 when a cluster of Lyme disease-like illnesses emerged in Minnesota and Wisconsin (1). *B. mayonii* infection appeared to be regionally specific, with cases only reported in the Upper Midwestern United States (North Dakota, Minnesota, and Wisconsin) (1–5). Further, *B. mayonii* is unique to other LD species due to several clinical presentations not typically associated with LD, including high spirochetemia (presence of high numbers of spirochetes in the blood), diffuse macular rashes, as well as nausea and vomiting. Since the 2016 study, only eight cases have been identified (1, 3, 4).

Spirochetemia was found in six patients, by a combination of quantitative PCR (5 patients; 4.2x10^5^ – 6.4x10^6^ copies of *oppA* per milliliter of blood), culture (2 patients, both positive), and/or microscopy of blood smears (2 patients, both positive) (1, 5). All six patients tested for spirochetemia had detectable spirochetes in their blood. Spirochetemia is not a typical sign associated with *B. burgdorferi* or other LD agent infection and is conventionally associated with relapsing fever (RF) (6, 7). The quantified number of *B. mayonii* spirochetes per milliliter of blood in patients is similar to levels seen in RF patients. *B. mayonii* spirochetemia levels in patients are 50 to 8000 times higher than what are seen in LD patients, typically between 10^2–^10^3^ cells per milliliter of blood, and even higher than patients infected with *B. miyamotoi*, with reported spirochetemia between 10^3–^10^5^ cells per milliliter of blood (7–12).

A few studies have demonstrated that *B. mayonii* is infectious for outbred and inbred laboratory mice, and that it disseminates to various tissues including urinary bladder, heart, spinal cord, ear, tibiotarsal joint, and meninges (13–15). The goal of this study was to directly compare both typical and atypical LD pathologies of *B. burgdorferi* and *B. mayonii* in a disease-susceptible host to identify differences that can be exploited for identification of bacterial virulence determinants and increased recognition for improved diagnoses.

## Methods

### Cultures and conditions

*B. mayonii* isolate BmnHA (host-passaged strain MN14-1539HA) and *B. burgdorferi* isolate B31-A3 were used in this study (14). Spirochetes were grown to mid-log-phase (1–5 x 10^7^ cells/mL) in BSK-II culture medium supplemented with 6% rabbit serum at 35°C under 5% CO2 and counted on a Petroff-Hausser counting chamber (16). For cultivation of blood and other mouse tissues, culture medium was supplemented with 50µg/mL rifampin, 20µg/mL phosphomycin, and 2.5µg/mL amphotericin B to prevent growth of contaminants.

Freezer stocks of *B. mayonii* and *B. burgdorferi* were made using glycerol as a cryoprotectant, with culture mixed 3:1 with 80% glycerol (final concentration 20% glycerol) then stored at -80°C. Cultures were started from freezer stocks by taking a scraping from the stock using a sterile inoculation loop then inoculating 5mL of fresh medium. Once this culture reached mid-log phase, it was sub-cultured to 10^5^ cells/mL in 5mL of fresh medium and then counted daily until the desired density for use was achieved.

### Animal care and use

Infection experiments were conducted according to the University of North Dakota Institutional Animal Care and Use approved protocol #2501–0001 and Institutional Biosafety Committee protocol #IBC2209-0010. Immunodeficient C3SnSmn.Cg-*Prkdcscic*/J (SCID; stock # 001131) and immunocompetent C3HeB/FeJ (C3H; stock # 000658) were purchased from the Jackson Laboratory (Sacramento, CA, USA). All experiments used 6–8 weeks old female mice that were housed based on infection groups. Animals were housed and cared for at the University of North Dakota Center for Biomedical Research. Food and water given ad libitum.

### Inoculations and blood sample collection

Mice were infected by needle-inoculation with 10^5^ cells/mouse in 100µL culture medium. Mice were anesthetized with isoflurane using a VetEquip Isoflurane Vaporizer (VetEquip, # 911103) and fur was removed from the back of the neck and scapular area using electric clippers. 70% ethanol was used to briefly disinfect the area. Needle-inoculation was intradermal in the upper thoracic midline. Mice were then returned to cage and monitored until anesthesia wore off.

Blood was collected from mice from the common iliac vein on alternating hind legs. Mice were placed head-first in a 50mL tube with the point cut off to allow airflow. The hind leg was held straight so hair could be shaved off the inner leg with a razor to expose the vein. The shaved area was briefly disinfected with 70% ethanol, dried, then a needle was used to perform venipuncture. Blood was collected with a pipette as it beaded up. Gauze was used to apply pressure until bleeding ceased. For experiments when blood was collected every other day, approximately 20µL of blood was collected: exactly 10µL blood was added to 90µL SideStep Lysis and Stabilizing Buffer (“lysis buffer”; Agilent, # 400900) and the tube flicked to mix, and approximately 10µL of blood was added to BSK-II for blood culture. For experiments when blood was collected once per week, exactly 20µL of blood was added to 180µL lysis buffer, and approximately 10µL of blood was added to BSK-II for blood culture. Blood in lysis buffer was stored at -80 °C for qPCR. Blood in BSK-II was incubated as per culture conditions. After 48 hours, media was aspirated without disturbing blood cells and transferred into a fresh tube with 500-750µL fresh BSK-II. Cultures were checked for spirochetes using darkfield microscopy for 8 weeks after collection.

### Tissue culture

To confirm that mice were infected with *B. mayonii* and *B. burgdorferi*, tissues were cultured. Blood was collected via puncture of the submandibular vein at 7 dpi, and 2mm ear punches (cleansed with 70% ethanol) from 2–4 weeks post-inoculation were cultured. At euthanasia, dura mater and whole ear were also cultured. Blood was sub-cultured after 48 hours. Tissues were sub-cultured after 7 days. Darkfield microscopy was used to assess cultures for spirochetes up to 8 weeks.

### Blood sample cleanup and detection of *B. mayonii* and *B. burgdorferi* using quantitative PCR

To reduce inhibition of PCR amplification due to blood products, blood in lysis buffer was column-cleaned using the Zymo *Quick*-DNA Microprep Kit according to the Whole blood, plasma, and serum protocol and eluted in 20µL elution buffer (Zymo Research, # D3021). For column-cleaning, samples were grouped by mouse with an uninfected blood sample processed alongside each group. Samples were stored at -20°C until use. qPCR was performed in 25µL reactions with 2µL DNA sample, *flaB* specific primers and probe, Bio-Rad SsoAdvanced Universal Probes Supermix (Bio-Rad, # 1725282). Forward primer GAGTTTCTGGTAAGATTAATGCTC. Reverse primer CATTTAAATTCCCTTCTGTTGTCTGA. Probe/56-FAM/AGAGGTTTG/ZEN/TCACAAGCTTCTAGAAATACTTCAAAGGC/3IABkFQ/. Samples were quantified in triplicate and copies per milliliter were calculated using the known quantity of blood per reaction, on the premise that one copy of *flaB* equates to one spirochete. Standard curves were created by amplifying known quantities of *flaB*. Standards were made by using primers to amplify *flaB* using PCR (14). Zero Blunt^®^ TOPO^®^ PCR Cloning Kit (ThermoFisher, # 450245) was used to clone PCR product into *E. coli* following manufacturer protocol. Plasmid DNA was purified using QIAprep^®^ Spin Miniprep Kit according to manufacturer’s protocol (Qiagen, # 27104). Copy numbers were quantified, aliquoted for standards, and stored at -80°C.

Spirochetemia in SCID mice was compared at 7, 14, 21 and 28 dpi in mice infected with *B. mayonii* and *B. burgdorferi* and statistical analysis was performed on the means of each timepoint using a two-tailed unpaired T-test with Welch’s correction. Additional statistical analyses are listed in text.

### Tibiotarsal joint measurements

Tibiotarsal joints were measured in millimeters using digital calipers (ULINE, # H-7352) while mice were under anesthesia with isoflurane. Mice were placed in a prone position and the widest part of the left and right tibiotarsal joints were measured and recorded, starting at time of inoculation (week 0) and then weekly until euthanasia or 4 weeks. After week 0, the measurer was blinded to the infection group and the same measurer was used across all experiments.

Statistics were performed on the mean and standard deviation of raw joint measurements by Two-way ANOVA with Šídák’s multiple comparisons test where uninfected mice were compared with *B. mayonii* and *B. burgdorferi* infected mice. Comparisons were made on the same sides (right vs right, left vs left) at the same time points (0 weeks vs 0 weeks; 1 week vs 1 week; etc).

### Histopathology

Tibiotarsal joints and hearts to be used for histopathology were briefly rinsed in PBS then submerged in 40mL of 10% neutral buffered formalin (Sigma-Aldrich, product #HT501128) and placed on a rocking plate at room temperature for 48 hours with the formalin solution replaced after 24 hours. Tibiotarsal joints required a brief decalcifying step in HCl prior to sectioning. Tissues were put into cassettes, mounted in paraffin, and cut on a Leica Paraffin Microtome (RM2125 RTS) in 3µm (heart) and 6µm (joint) sections. Hearts were sectioned along the frontal plane. Joints were sectioned along the lateral plane. Representative sections for each sample were stained with hematoxylin and eosin for visualization with light microscopy. All histopathology scoring was done by an ACVP board certified veterinary pathologist who was blinded to the identity of the samples. Representative images tissues were scanned with a 40x objective using an Epredia Pannoramic MIDI II slide scanner.

Heart histopathology was completed on 50 mice (20 infected with *B. mayonii*, 20 infected with *B. burgdorferi*, and 10 vehicle/media only). Joint histopathology was completed on 25 mice (10 infected with *B. mayonii*, 10 infected with *B. burgdorferi*, and 5 vehicle/media only) due to complications with decalcification on the first 25 mice.

### Statistics

Statistical analyses were performed using GraphPad Prism 10.5.0.

## Results

### Spirochetemia in mice

The presence of bacteria in the blood is referred to as bacteremia, and for spirochetes, spirochetemia. *Borrelia* species that make up the *Borrelia burgdorferi sensu lato* (*Bbsl*) complex are rarely detectable in the blood (7). Reports on successful cultivation of *B. burgdorferi* sensu stricto from the blood or serum of patients varies, with success rates between 5% and 40% reported, and spirochetes are not readily visualized by microscopy (8). Experimental studies in laboratory animals suggest *B. burgdorferi* exists only transiently in the blood and at low densities (17–19).

In a previous study, we were unable to culture *B. mayonii* from the blood of C3H mice at 7 dpi (14) however, other timepoints were not examined. In severe combined immunodeficient (SCID) mice, *Bbsl* species reach higher levels of spirochetemia that is maintained and readily detectable by culture and qPCR (20). Therefore, we first tested if *B. mayonii* was detectable in SCID mice by culture and qPCR.

**Figure 1:**
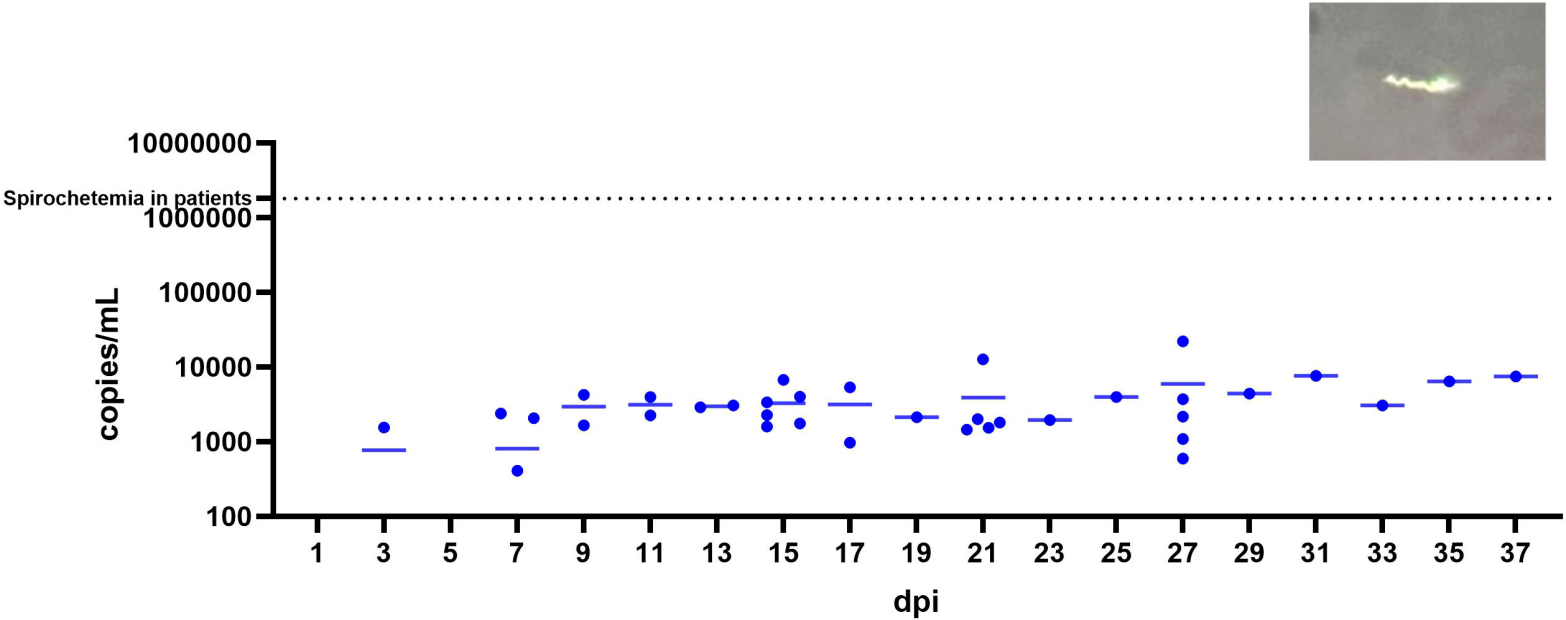
*B. mayonii* spirochete in the plasma of a SCID mouse at ~6 weeks post-inoculation, darkfield microscopy (400X). qPCR data of *B. mayonii* spirochetemia in SCID mice (n=7) between 3–37 dpi. Each point represents one blood sample; blood samples were not collected from all mice at all timepoints. Bars indicate mean spirochetemia at each timepoint; samples with no detectable spirochetes were included in mean calculations, though points are not shown on graph. X-axis is days post-inoculation. Y-axis is copies of flaB per mL of blood. A horizontal dotted line indicates average spirochetemia in human patients with *B. mayonii* infection.

A total of seven SCID mice were infected with *B. mayonii* across three experiments with blood samples en masse collected from 3–37 dpi (**Figure 1**) for blood culture and qPCR. The earliest timepoint tested for culture positivity was 7 dpi and the latest was 37 dpi. All blood cultures from SCID mice infected with *B. mayonii* were positive between 7–37 dpi, indicating that *B. mayonii* is present in the blood of SCID mice and can be cultured from blood. All mice developed similar levels of spirochetemia, with spirochetemia detectable by qPCR as early as 3 dpi. Across the infection, *B. mayonii* spirochetemia appears to plateau from 9–37 dpi, ranging from 5.92x10^2^ – 2.21x10^4^ copies of *flaB* per milliliter of blood, with an average density of 4.07x10^3^ copies per milliliter. Spirochetes were occasionally visible in plasma by darkfield microscopy (inset, **Figure 1**).

**Figure 2:**
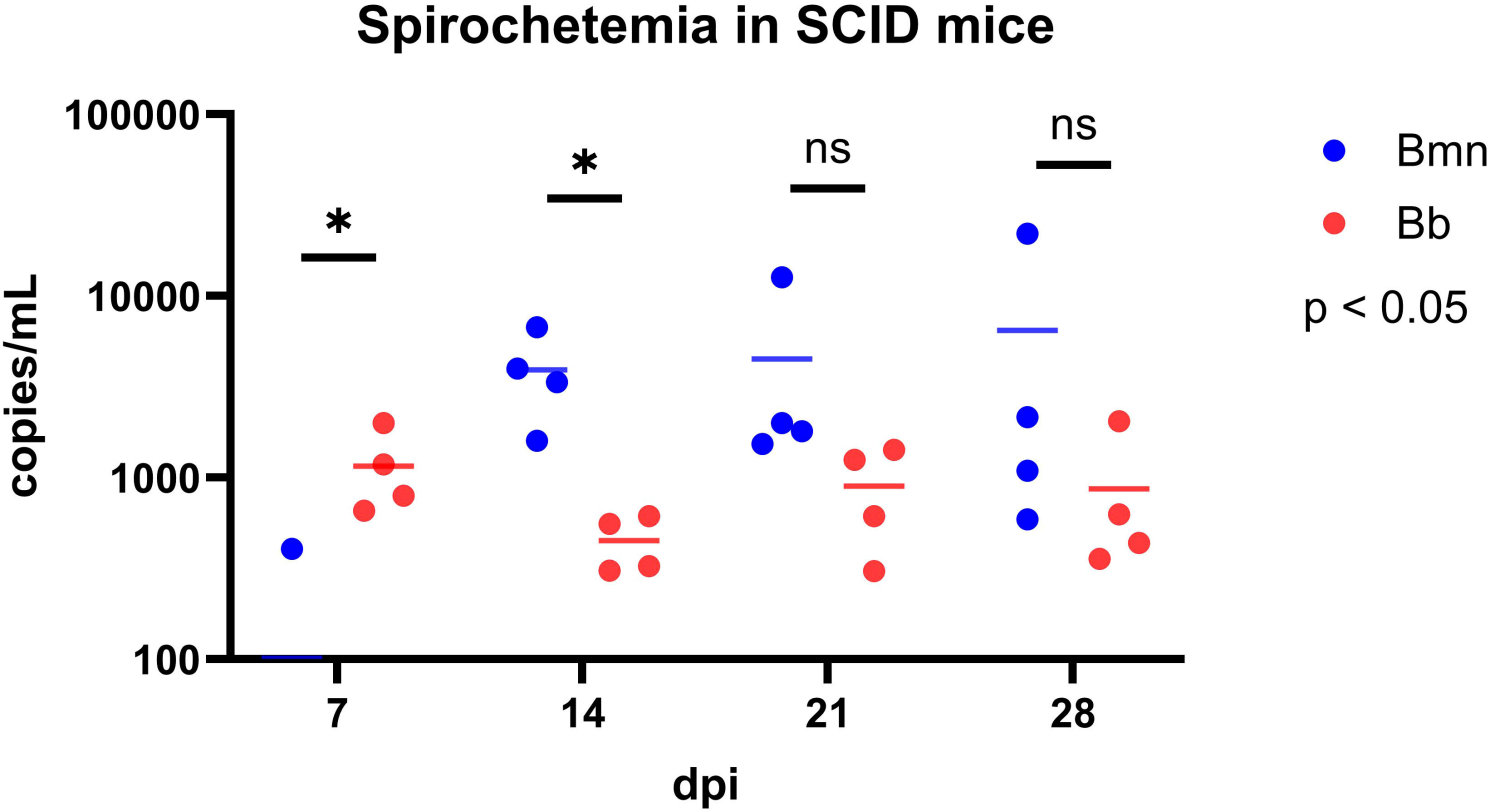
qPCR data of *B. mayonii* (blue dots) and *B. burgdorferi* (red dots) with mean spirochetemia as a bar in SCID mice at 7, 14, 21, and 28 dpi (n=4 per infection group). At 7 dpi, three *B.* mayonii-infected SCID had undetectable levels of spirochetes in the blood, thus only one dot is present with no mean bar. X-axis is days post-inoculation. Y-axis is copies of flaB per mL of blood. Unpaired t test with Welch’s correction was used to compare means at each timepoint. 7 dpi p=0.0333. 14 dpi p=0.0468. 21 dpi p=0.2782. 28 dpi p=0.3606. * p<0.05, ns, not significant.

We then compared spirochetemia from SCID mice infected with *B. mayonii* or *B. burgdorferi* at 7, 14, 21, and 28 dpi for culture and qPCR (**Figure 2**). All blood cultures from all SCID mice were positive. An interesting observation was that the 7 dpi blood cultures for *B. mayonii* took two days longer than *B. burgdorferi* to become positive, suggesting that *B. mayonii* were at lower levels in the blood at 7dpdi. qPCR data supported this, showing that at 7dpi *B. burgdorferi* infected SCIDs had significantly higher spirochetemia than *B. mayonii* infected SCIDs (P = 0.0333). However, at 14 dpi *B. mayonii* has significantly higher spirochetemia (P = 0.0468). By 21 and 28 dpi, spirochetemia appears to have leveled off, with *B. mayonii* spirochetemia trending slightly higher, though non-significant (21 dpi P = 0.2782; 28dpi P = 0.3606; Unpaired t test with Welch’s correction).

Spirochetemia was also measured in immunocompetent C3H mice at 7, 14, 21, and 28 dpi. There were only two positive blood cultures from the same *B. mayonii* infected C3H mouse at 7 and 14 dpi. On the contrary, *B. burgdorferi* infected mice had positive blood cultures at all timepoints from at least one mouse. There was no significant difference between *B. mayonii* and *B. burgdorferi* in the number of positive blood cultures per mouse (**Table 1**; Mann Whitney rank test; P = 0.4857).

**Table 1:** From C3H mice infected with *B. mayonii* (n=4) and *B. burgdorferi* (n=4), blood samples were collected from each mouse at 7, 14, 21, and 28 dpi, for a total of 16 blood samples per infection group.

	*B. mayonii*		*B. burgdorferi*	
Week	Blood Culture	qPCR	Blood Culture	qPCR
1	1/4	0/4	1/4	0/4
2	1/4	0/4	1/4	1/4
3	0/4	0/4	2/4	0/4
4	0/4	0/4	1/4	1/4

Blood samples were tested for the presence of spirochetes with blood culture and qPCR with the table showing the number of positive samples at each timepoint out of the total samples collected. Of the blood samples collected from *B. mayonii* and *B. burgdorferi* infected C3H mice for blood culture two total cultures and five total cultures were positive, respectively. Mann Whitney rank test; p=0.4857. Of the blood samples collected from *B. mayonii* and *B. burgdorferi* infected C3H mice for qPCR analysis, zero total cultures and two total cultures had qPCR amplification, respectively. Statistical analysis was not performed.

All blood samples from C3H mice infected with *B. mayonii* were qPCR negative from 7–28 dpi. Two blood samples from *B. burgdorferi* infected mice were qPCR positive (**Table 1**). Statistical analysis was not performed. To test if *B. mayonii* takes longer than 28 days to reach detectable levels in the blood, two additional C3H mice had blood collected every other day from 19–37 dpi, all of which were qPCR negative (blood was not cultured). Infection was confirmed in mice with culture of joint, ear, or dura (data not shown).

### *Borrelia mayonii* tibiotarsal joint pathology

In C3H mice infected with *B. burgdorferi*, edema and pathology of the tibiotarsal joints peaks between 3–4 weeks post-infection (21). This pathology can be assessed by measuring joints for swelling and by using histopathology to assess the severity of arthritis. Therefore, this mouse model is best suited to investigating acute Lyme arthritis, whereas other strains are better suited to studying antibiotic refractory Lyme arthritis or post-treatment Lyme disease syndrome (22).

Of the eight confirmed cases of *B. mayonii* infection in patients, site-specific arthralgia was only reported in one patient (left knee), though diffuse arthralgias and myalgias were reported in seven patients (1, 3, 4). This may indicate that *B. mayonii* is only minimally arthritogenic, or simply that patients presenting with other symptoms like fever, headache, and diffuse macular rashes received antibiotic treatment prior to the onset of arthritis.

**Figure 3:**
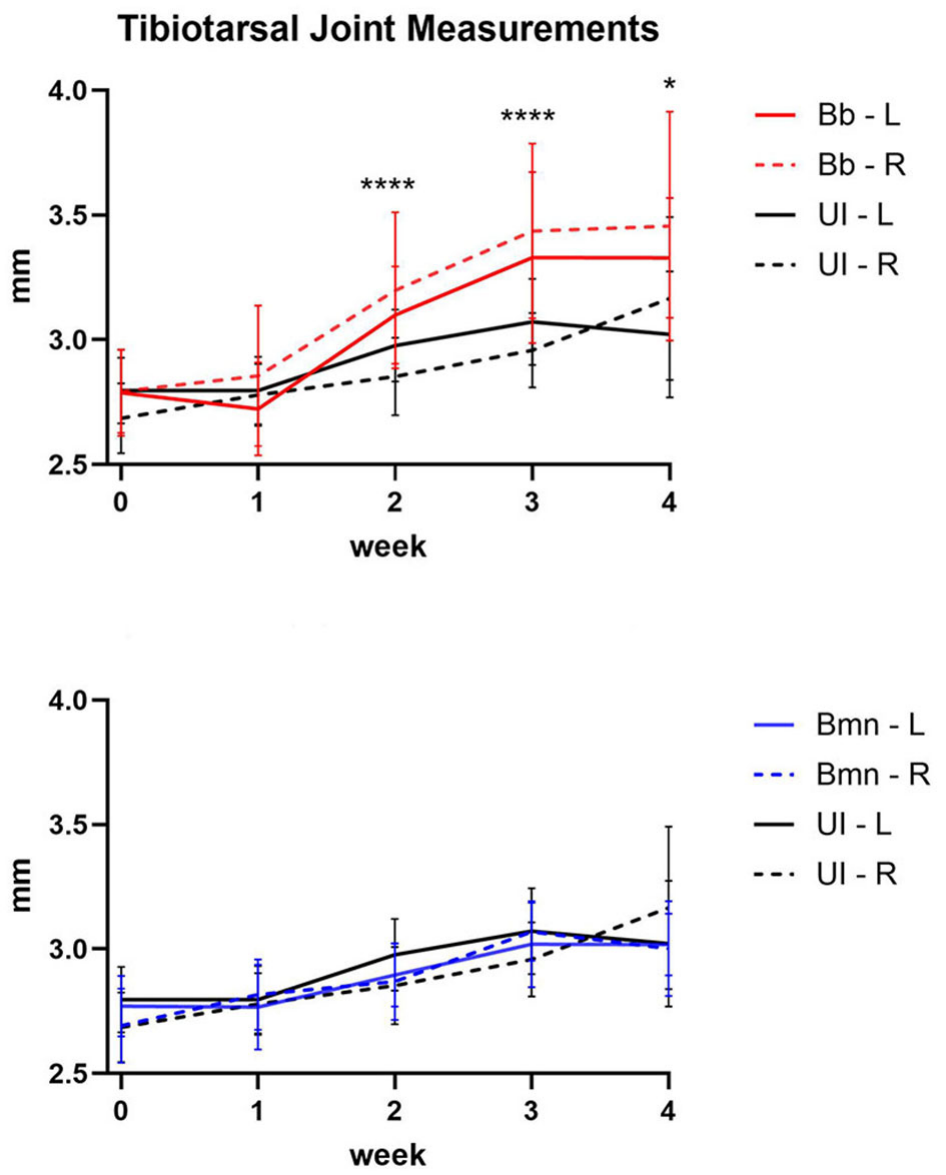
Average measurements of tibiotarsal joints with error bars (L – left, solid lines; R – right, dashed lines) of C3H mice infected with *B. burgdorferi* (Bb, red), or media only (UI, black; panel **A**) or *B. mayonii* (Bmn, blue), or media only (UI, black) in millimeters from 0–4 weeks (Statistics are shown in **Table 2**. The same uninfected measurements are used for both comparisons. **(A)** comparison of means of tibiotarsal joints measurements from Bb infected mice (0–2 weeks n=20; 3–4 weeks n=10) with uninfected mice (0–2 weeks n=12; 3–4 weeks n=10). **(B)** comparison of means of tibiotarsal joints measurements from Bmn infected mice (0–2 weeks n=27; 3–4 weeks n=14) with uninfected mice (0–2 weeks n=12; 3–4 weeks n=10). * p<0.05; **** p<0.001.

**Figure 4:**
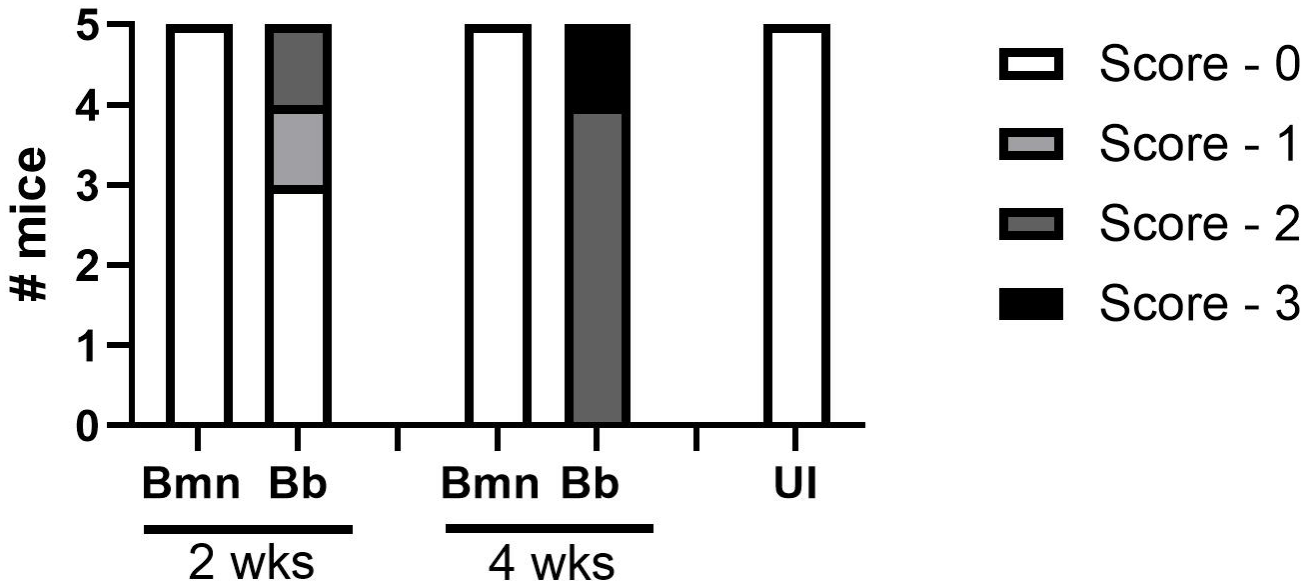
Tibiotarsal joint sections from *B. mayonii* infected (n=5 at 2 weeks, n=5 at 4 weeks), *B. burgdorferi* infected (n=5 at 2 weeks, n=5 at 4 weeks), or uninfected mice (n=5 at 4 weeks) were blindly scored by a board certified ACVP veterinary pathologist (see Methods, Histopathology). X-axis groups the Bmn and Bb mice at 2 and 4 weeks and the UI mice on their own. Y-axis is number of mice. Each bar represents 5 mice and their scoring at a timepoint and condition as parts of a whole, with the shading indicating the severity of the histopathology scoring, the lightest being no pathology and the darkest being the most pathology. At 2 weeks all 5 Bmn infected mice received scores of 0, while of the Bb infected mice three received scores of 0, one received a score of 1, and one received a score of 2. At 4 weeks all 5 Bmn infected mice received scores of 0, while of the Bb infected mice four received scores of 2 and one received a score of 3. All five UI mice received scores of 0.

**Figure 5:**
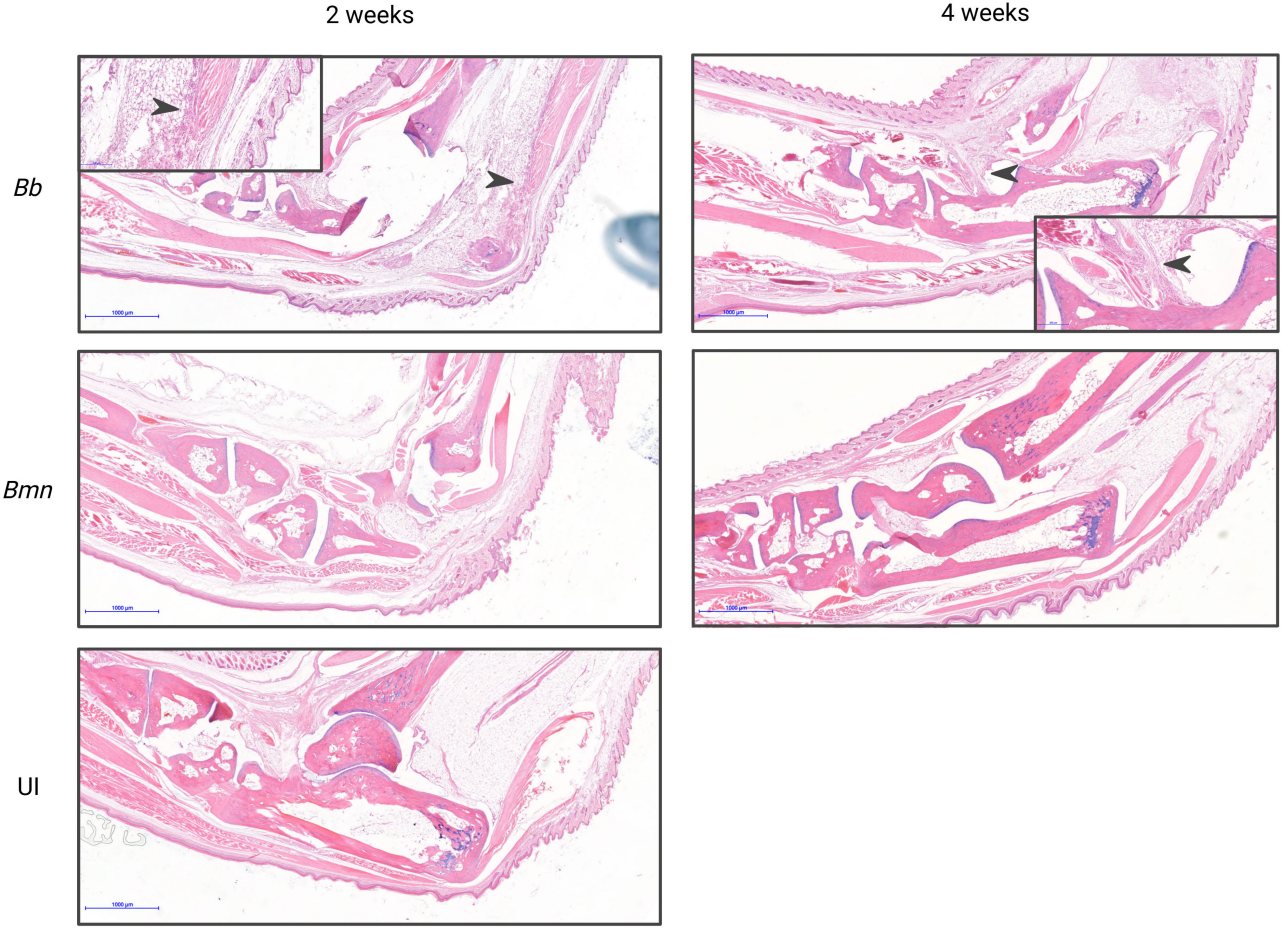
Photomicrographs of tibiotarsal joints stained with H&E from mice infected with *B. mayonii*, *B. burgdorferi*, or uninfected at 2 and 4 weeks post-inoculation. All representative images shown at 2X, with inset panels highlighting pathology at 10X. Bb at 2 weeks 10X inset has an arrow indicating tendinitis. *B. burgdorferi* at 4 weeks 10X inset has an arrow indicating synovitis, periarticular fat, and marked edema. *B. mayonii* at 2 weeks, *B. mayonii* at 4 weeks, and UI demonstrate no histopathology.

**Figure 6:**
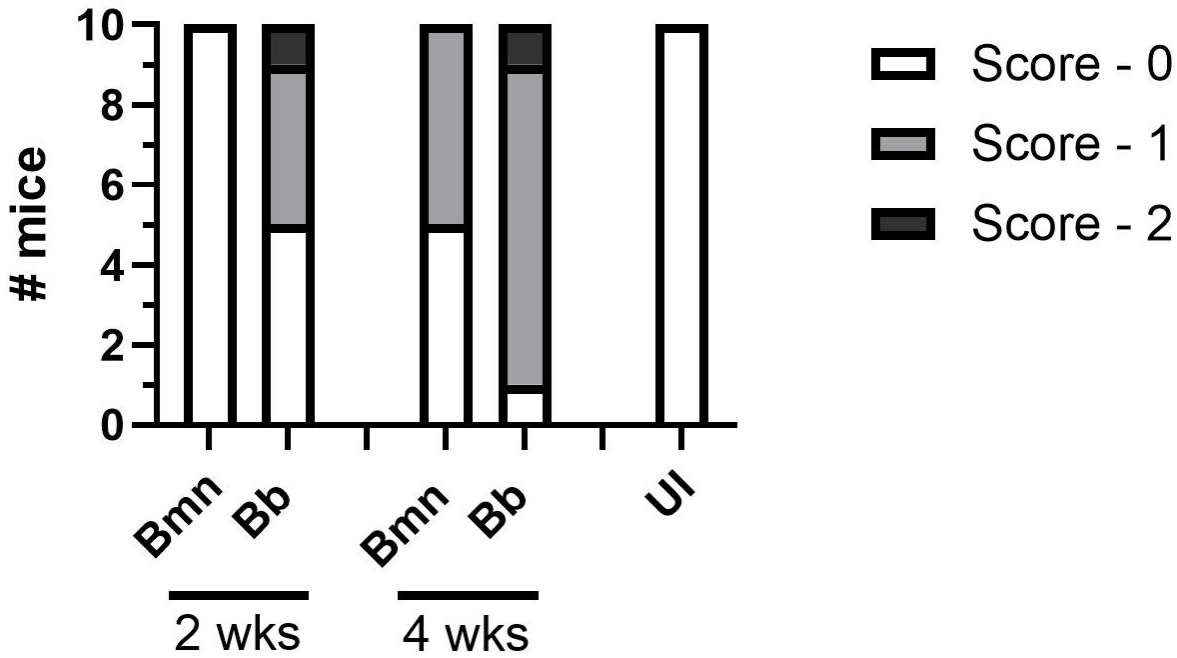
Heart sections from *B. mayonii* infected (n=10 at 2 weeks, n=10 at 4 weeks), *B. burgdorferi* infected (n=10 at 2 weeks, n=10 at 4 weeks), or uninfected mice (n=10 at 4 weeks) were blindly scored by a board certified ACVP veterinary pathologist (see Methods, Histopathology). X-axis groups the Bmn and Bb mice at 2 and 4 weeks and the UI mice on their own. Y-axis is number of mice. Each bar represents 10 mice and their scoring at a timepoint and condition as parts of a whole, with the shading indicating the severity of the histopathology scoring, the lightest being no pathology and the darkest being the most pathology. At 2 weeks all 10 Bmn infected mice received scores of 0, while of the Bb infected mice five received scores of 0, four received scores of 1, and one received a score of 2. At 4 weeks 5 Bmn infected mice received scores of 0 and five received scores of 1, while of the Bb infected mice one received a score of 0, eight received a score of 1, and one received a score of 2. All ten UI mice received scores of 0.

**Figure 7:**
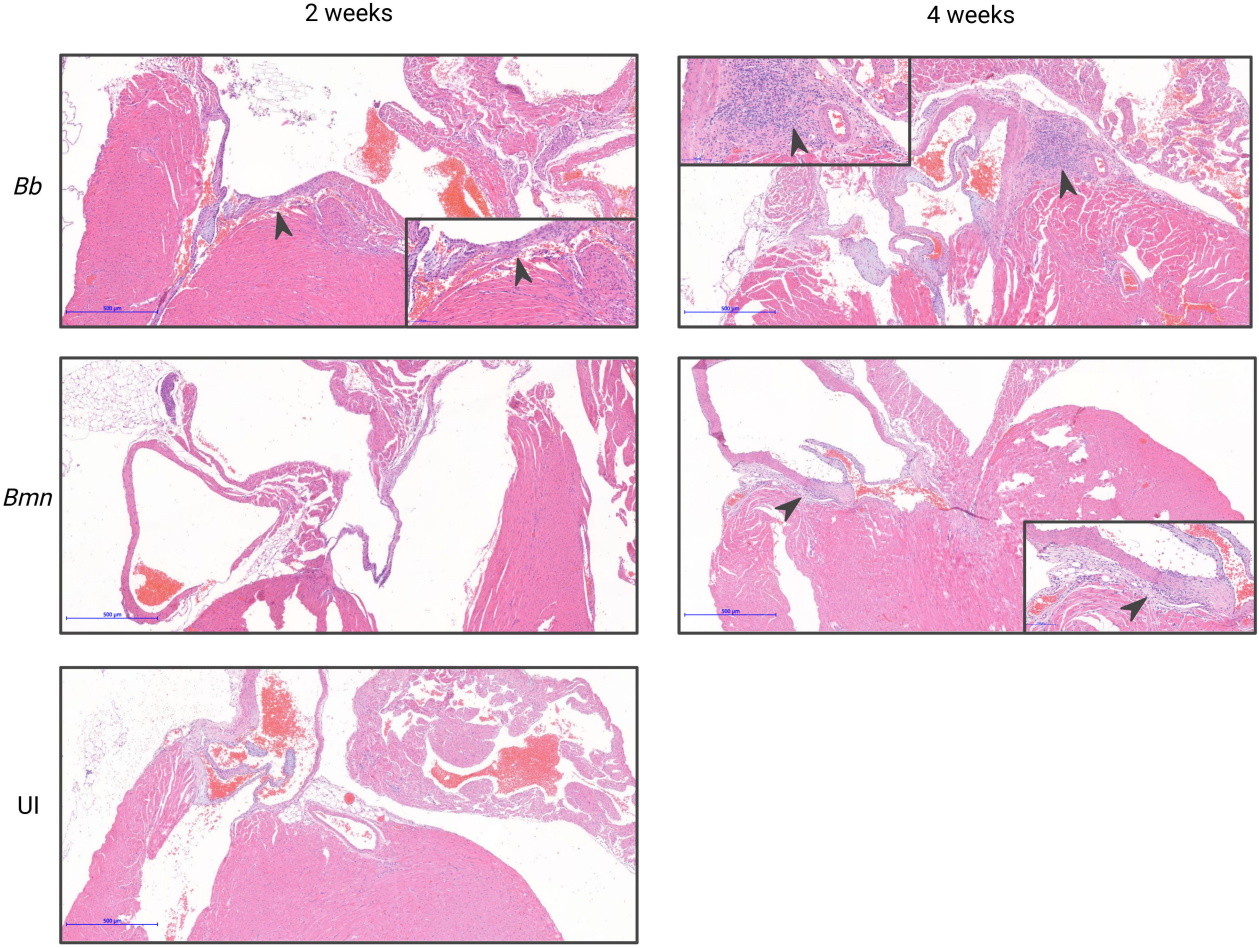
Photomicrographs of hearts stained with H&E from mice infected with *B. mayonii*, *B. burgdorferi*, or uninfected at 2 and 4 weeks post-inoculation with veterinary histopathologist score and notes. All representative images at 5X, with insets at 20X highlighting pathology. *B. burgdorferi* at 2 weeks 20X inset with an arrow indicating infiltrates, perivalvular, perivascular, mixed mononuclear, regionally extensive, and minimal. *B. burgdorferi* at 4 weeks 20X inset with an arrow indicating infiltrates, perivalvular, perivascular, mixed mononuclear, multifocal, moderate with perivalvular and perivascular myocarditis. *B. mayonii* at 4 weeks 20X inset with an arrow indicating infiltrates, perivalvular, perivascular, mixed mononuclear, regionally extensive, and mild. *B. mayonii* at 2 weeks and UI demonstrate no histopathology.

To determine if *B. mayonii* infection produces arthritis in C3H mice, demonstrated by joint swelling and histopathology, C3H mice were inoculated either with *B. mayonii*, *B. burgdorferi*, or media/vehicle only for uninfected controls. Rear tibiotarsal joints were measured with digital calipers at the time of infection (0 weeks), and weekly after that until euthanasia (**Figure 3**). The infection group was blinded to the measurer starting at one week post-inoculation. At 2 and 4 weeks post-inoculation mice were euthanized and rear tibiotarsal joints and hearts were fixed for histopathology (**Figures 4**–**7**).

Šídák’s multiple comparisons test was used to compare the means of uninfected mouse joint size with the means of *B. mayonii* and *B. burgdorferi.* There was no statistically significant difference in joint sizes between *B. mayonii* infected and uninfected mice. *B. burgdorferi* infected mice developed monoarticular joint swelling on the right side with significantly larger tibiotarsal joints at 2, 3, and 4 weeks post-inoculation (p<0.0001, p<0.0001, p=0.025, respectively), and on the left side at 4 weeks post-inoculation (p=0.0128) (**Figure 3**, **Table 2**). Comparisons were made on the same side (left vs. left; right vs. right) at the same timepoints (0 vs. 0; 1 weeks vs. 1 week; etc).

**Table 2:** Statistics from **Figure 3**.

Uninfected vs.	*B. mayonii*		*B. burgdorferi*		*n*
*Week*	*Left*	*Right*	*Left*	*Right*	
0	ns, p>0.9999	ns, p>0.9999	ns, p>0.9999	ns, p=0.9524	Bmn = 27; Bb = 20; UI = 12
1	ns, p>0.9999	ns, p>0.9999	ns, p>0.9999	ns, p>0.9999	
2	ns, p=0.9962	ns, p>0.9999	ns, p=0.8545	****, p<0.0001	
3	ns, p>0.9999	ns, p=0.9801	ns, p=0.0788	****, p<0.0001	Bmn = 14; Bb = 10; UI = 10
4	ns, p>0.9999	ns, p=0.6146	*, p=0.0128	*, p=0.025	

Šídák’s multiple comparisons test; comparisons were made between same sides only (L vs. L; R vs. R)

Histopathology supported joint measurement data. Joints were scored at 2 and 4 weeks post-inoculation on inflammation within periarticular tissues: 0=None, 1=Focal to multifocal and minimal to mild (<5% of the total section), 2=Focal to multifocal and moderate (5-10% of the total section), 3=Multifocal and moderate to marked (11-25% of the total section), 4=Multifocal to coalescing and marked (>25% of the section). Mice infected with *B. mayonii* had no histopathology scoring at 2 weeks (0/5 mice) or 4 weeks (0/5 mice) post-inoculation. Comparatively, *B. burgdorferi* infected mice had scoring at 2 weeks (2/5 mice) and 4 weeks (5/5 mice), with notes of edema and synovitis at 4 weeks (**Figures 4**, **5**). Infection was confirmed by tissue culture in *B. mayonii* infected mice (9/10 mice) and *B. burgdorferi* infected mice (10/10 mice).

Based on measurable joint swelling and histopathology, *B. mayonii* infected C3H mice do not develop Lyme arthritis within 4 weeks post-inoculation.

### Borrelia mayonii heart pathology

It is unknown if *Borrelia mayonii* infection results in Lyme carditis in patients. One patient reported shortness of breath combined with dry cough, and six other patients reported weakness (1, 3, 5). With the limited number of confirmed cases and no confirmed diagnoses of Lyme carditis, it is unclear if these symptoms are suggestive of cardiac infiltration or the result of other pathologies.

Published literature confirms *B. mayonii* colonization of C3H cardiac tissues by culture and qPCR, but only at 28 dpi (14). In *B. mayonii* infected CD-1 mice, hearts were all culture negative and intermittently PCR positive between 163–375 dpi (15). No studies have investigated pathology associated with cardiac colonization by *B. mayonii*.

For this study to determine if *B. mayonii* infection results in cardiac pathology, C3H mice were inoculated with either *B. mayonii*, *B. burgdorferi*, or media only for uninfected controls. Mice were euthanized at 2 or 4 weeks post-inoculation and hearts were collected and prepared for histopathology. Heart tissues were stained with H&E, blinded, and scored as 0 = No pathology, 1 = Focal/regional extensive and minimal to mild (<10% of the total section), 2 = Focal/regional extensive and moderate (10-25% of the total section), 3 = Multifocal and moderate (26-50% of the total section), 4 = Multifocal to coalescing and marked (>50% of the section). Pathology scores reflect focal to multifocal inflammatory lesions, including valvular endocarditis, histiocytic and neutrophilic infiltrates, and perivascular lymphoplasmacytic inflammation.

At 2 weeks post-inoculation *B. mayonii* infected C3H mice demonstrated no pathology, evidenced by all slides being scored 0, with no evidence of cellular infiltration or associated inflammation (0/10 mice). At 4 weeks post-inoculation half of all *B. mayonii* infected mice demonstrated carditis (5/10 mice), with scores of 1 and the pathologist noting perivascular and perivalvular infiltration and myocarditis. Half of *B. burgdorferi* infected mice presented with histopathology at 2 weeks post-inoculation (5/10 mice) and 90% of mice had pathology at 4 weeks post-inoculation (9/10 mice), with majority of scoring being 1, with minimal scores of 2 (**Figures 6**, **7**). This indicates that *B. mayonii* may be colonizing the heart and surrounding tissues more slowly than *B. burgdorferi* and that carditis is somewhat less severe, evidenced by scoring. However, this confirms that *B. mayonii* colonization of the heart has the potential to lead to Lyme carditis.

## Discussion

In this study, we demonstrated that *B. mayonii* did not reliably cause detectable spirochetemia in C3H mice up to 37 days post-inoculation. These results are consistent with previous studies in which *B. mayonii* was undetectable by culture or qPCR from the blood (14, 15). However, in SCID mice where spirochetemia was reliably detected, levels were still far lower than reported levels in immunocompetent human patients. Patients had spirochetemia ranging from 4.2x10^5^ – 6.4x10^6^ copies of *oppA* per milliliter of blood with an average of 1.81x10^6^ copies per milliliter, 100X – 1000X higher than levels demonstrated in SCID mice (**Figure 1**). This suggests that *B. mayonii* may have human-specific mechanisms for survival in the blood or that *B. mayonii* is uniquely susceptible to components of mouse blood. An important consideration with the observed spirochetemia in human patients is the low number of confirmed cases; are atypical symptoms the standard or an artifact of sampling bias? An interesting observation was that both blood culture and qPCR demonstrated that *B. mayonii* took 2 additional days to reach the blood of SCID mice compared with *B. burgdorferi*, suggesting that *B. burgdorferi* either disseminates to the blood more rapidly and/or once in the blood begins replicating more quickly than *B. mayonii*.

*B. mayonii* did not induce observable joint swelling or pathology in C3H mice, though *B. mayonii* can be routinely cultured and detected by qPCR in the joint (14). This indicates that while *B. mayonii* are present in the joints of C3H mice, the host immune response is muted or delayed, potentially similar to mouse infections with *B. afzelii* (23). Alternatively, it could be possible that *B. mayonii* induces arthritis in the mouse on a different timeline from *B. burgdorferi*, with swelling and pathology only occurring after months of infection. As *B. mayonii* has a reduced genome compared to *B. burgdorferi*, this likely contributes to differences in pathology associated with C3H mice (2).

The results of this study also demonstrate that *B. mayonii* infection coincides with cardiac pathology, particularly around valves, blood vessels, and other connective cardiac tissue in half of infected animals at 4 weeks post-inoculation. However, at 2 weeks post-inoculation *B. mayonii* infected mice did not present with cardiac pathology (0/10 mice), where *B. burgdorferi* infected mice did (5/10 mice), further indicating that *B. mayonii* may have a dissemination defect that increases the incubation time prior to clinical presentations. *B. mayonii*, like *B. burgdorferi*, persistently infects mice, and future studies should investigate later timepoints for carditis, and determine resolution and potential inflammatory flareups (15). Therefore, *B. mayonii* has the potential to cause Lyme carditis.

## Conclusions

To our knowledge, this is the first study to examine *B. mayonii* pathogenesis in a mouse model. Consistent with other Lyme borreliae, *B. mayonii* causes persistent infection in a C3H model, though there are distinct differences in immunopathology compared with *B. burgdorferi* infection. In contrast to reported human causes, however, we did not observe enhanced spirochetemia compared to *B. burgdorferi* sensu stricto. *B. mayonii* also failed to induce measurable arthritis but did induce mild carditis. Further studies are needed to examine *B. mayonii*-induced pathology at later timepoints and in different tissues such as the CNS. This model can be leveraged to investigate bacterial factors responsible for differences in immunopathologies and to better understand LD host-pathogen interactions. Additionally, our studies demonstrate that *B. mayonii* has a dissemination defect compared with *B. burgdorferi*, at least in mice, and clinicians should take this into account when diagnosing potential tick-borne illnesses.

## Data Availability

The raw data supporting the conclusions of this article will be made available by the authors, without undue reservation.
